# Skin autofluorescence in acute kidney injury

**DOI:** 10.1186/s13054-017-1598-0

**Published:** 2017-02-09

**Authors:** Aurelie Lavielle, Sebastien Rubin, Alexandre Boyer, Karine Moreau, Kalina Rajaobelina, Christian Combe, Vincent Rigalleau

**Affiliations:** 1 Endocrinologie-Nutrition, Université de Bordeaux, 33000 Bordeaux, France; 2 Néphrologie-Transplantation-Dialyse, Université de Bordeaux, 33000 Bordeaux, France; 3 Réanimation médicale, Université de Bordeaux, 33000 Bordeaux, France; 4 CHU de Bordeaux, Bordeaux Public Health, Université de Bordeaux, 33000 Bordeaux, France; 5 Unité INSERM 1026, Université de Bordeaux, Bordeaux, France; 6 Endocrinologie-Nutrition, CHU de Bordeaux, Avenue Magellan, 33600 Pessac, France

We were interested by the article from De Corte et al. about the poor long-term outcome after acute kidney injury (AKI) [1]. Besides initial oliguria, the three predictors for dialysis dependence were age, diabetes, and chronic kidney disease (CKD), which have previously been related to the accumulation of advanced glycation end-products (AGEs) as evaluated by skin autofluorescence (sAF). sAF is an indirect marker that has been related to the skin concentrations of fluorescent (pentosidine) and non-fluorescent AGEs (carboxy-methyl-lysine and carboxy-ethyl-lysine) in skin biopsies of hemodialized subjects [2]. Could sAF be altered in AKI?

From July 2014 to April 2015, we measured sAF with an AGE-Reader (DiagnOpticsTechnologies B.V., Groningen, Netherlands) in 35 patients admitted for AKI, staged F (RIFLE). Their results were compared to their theoretical values ((0.024 × Years of age) + 0.83 [3]) and to those of 35 patients with CKD waiting for a renal graft. All the patients gave written informed consent and the study was approved by the Comité de Protection des Personnes Sud-Ouest et Outre-Mer 3 (Bordeaux). A multivariate linear regression analysis was performed to study the relationship between sAF and the duration of renal failure and to adjust it to the age and gender of the subjects.

The patients with AKI and CKD had similar age, gender, body-mass index, and creatinine levels (Table 1). The sAF was lower in AKI than CKD, still significant (*p* < 0.001) after adjustment for age, gender, and creatinine. The sAF were higher than the theoretical values calculated from age: 2.31 ± 0.36 arbitrary units (AU; *p* < 0.001 for both AKI and CKD). The sAF was related to the duration of renal failure and was still significant (B = +0.43, *p* = 0.02) after adjustment for age and gender (Fig. 1). In six patients with AKI, a second sAF measurement was performed10 ± 3 days later: the sAF increased from 2.61 ± 0.72 to 3.03 ± 0.74 (*p* < 0.05).

**Table 1:** Characteristics of the subjects with acute and chronic renal failure

	Acute kidney injury	End-stage renal disease	*p*
N	35	35	
Age (years)	62 ± 15	62 ± 10	NS
Gender (percentage of women)	57%	54%	NS
Diabetes	31%	34%	NS
BMI (kg/m^2^)	26.5 ± 7.7	24.7 ± 3.8	NS
Serum creatinine (mg/dL)	7.0 ± 3.6	7.1 ± 1.7	NS
Duration of renal failure (days)	34 ± 28	3275 ± 2114	<0.001
Smokers	25%	28%	NS
sAF (AU)	2.97 ± 0.72	3.70 ± 0.72	<0.001

**Fig. 1:**
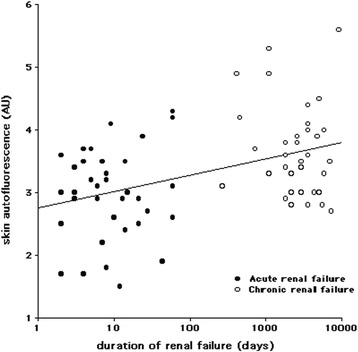
Skin autofluorescence according to the duration of renal failure

Our results show that sAF is lower in AKI than in CKD and relates to the duration of renal failure, as expected. sAF is considered as a marker of metabolic memory [4], reflecting the accumulation of AGEs in the skin [2]. In our patients who could be analyzed twice, sAF increased by +0.4 AU after only 10 days, so they probably had normal sAF when their AKI started one month before. A normal, early measured sAF may therefore help to distinguish acute from chronic renal failure. The sAF was already high, and increased rapidly, in our patients with AKI. This concurs well with the quick rising plasmatic concentrations of AGEs in experimentally induced acute renal failure in rats [5]. High sAF has also been reported in patients admitted to intensive care units [6], attributed to acute oxidative stress, which occurs in AKI. Although sAF is an indirect marker and fluorescent compounds other than AGEs accumulate in uremia, our results raise the hypothesis that the accumulation of AGEs during AKI may play a role in a later adverse outcome.

## Abbreviations

AGE: Advanced glycation end product
AKI: Acute kidney injury
BMI: Body mass index
CKD: Chronic kidney disease
sAF: Skin autofluorescence
